# Diagnostic Performance of the PrevoCheck for the Detection of Human Papillomavirus 16‐Driven Head and Neck Squamous Cell Carcinoma

**DOI:** 10.1111/jop.70075

**Published:** 2025-11-14

**Authors:** Charlotte S. Schouten, Vittoria Guarda, Thomas M. Stadler, J. Kristian Ikenberg, Martina A. Broglie Däppen

**Affiliations:** ^1^ Department of Otorhinolaryngology – Head and Neck Surgery University Hospital Zurich Zurich Switzerland; ^2^ Department of Pathology and Molecular Pathology University Hospital Zurich Zurich Switzerland

**Keywords:** HPV antibodies, HPV oncoproteins, HPV16, HPV‐related oropharyngeal cancer, oropharyngeal squamous cell carcinoma

## Abstract

**Background:**

Human papillomavirus (HPV)‐16 is the most commonly found HPV‐type in HPV‐induced oropharyngeal squamous cell carcinomas (OPSCC). The serological response to HPV oncoproteins could be a way to detect HPV‐driven OPSCC early. A rapid test for the detection of HPV16 L1 antibodies in blood was developed in 2015 (PrevoCheck).

**Methods:**

Prospectively, we included 42 patients with newly diagnosed head and neck squamous cell carcinomas (HNSCC). Pretreatment venous blood samples were collected and analyzed with PrevoCheck. The results were interpreted by 2 reviewers. Immunohistochemistry with p16 and HPV DNA‐PCR testing served as a reference standard.

**Results:**

Sixteen patients had HPV‐positive tumors (38.1%). PrevoCheck showed 2 true positives, 26 true negatives, 0 false positives, and 14 false negatives, which resulted in a sensitivity of 12.5% (95% CI: 1.6%–38.4%) at a specificity of 100% (95% CI: 86.8–100). Interobserver agreement showed perfect agreement.

**Conclusion:**

A negative result in a test with a high sensitivity can be used to rule out disease, that is, HPV16‐related HNSCC. We found 14 false negative results, resulting in low sensitivity for PrevoCheck. This test does not seem suitable to screen for HPV16‐related head and neck cancers.

## 1 Introduction

The incidence of human papillomavirus (HPV)‐related oropharyngeal squamous cell carcinoma (OPSCC) has increased significantly in the last decades. This increase is well documented in many economically developed countries such as the United States and Europe [1, 2, 3, 4]. Infection with a high‐risk HPV type can lead to the development of oropharyngeal cancer, with the tonsils having a higher prevalence than other oropharyngeal subsites. HPV16 is most commonly found in HPV‐induced OPSCC [5, 6].

HPV testing using tumor tissue is part of the routine diagnostic work‐up in OPSCC. The gold standard for detecting HPV involvement is a test algorithm using p16 immunohistochemistry as a surrogate marker, followed by HPV DNA PCR testing on the p16‐immunopositive cases. The patients that are positive in both assays are classified as HPV‐positive [7].

Stage at diagnosis is an important parameter, as it mostly determines the treatment strategy and prognosis. Unfortunately, most patients with head and neck squamous cell carcinoma (HNSCC) present with advanced staged disease after becoming symptomatic, especially patients with oropharyngeal cancer. About two thirds of the HPV‐induced OPSCCs are diagnosed as late‐stage tumors [8]. In the last decade, it has been reported that the serological response to the HPV oncoproteins E6 and E7 is associated with HPV‐driven OPSCC and the measurement of serum antibodies might be a mode to detect HPV‐driven oropharyngeal tumors earlier [9, 10].

In 2015, an immunoassay in the form of a rapid test for the qualitative detection of HPV16 L1 antibodies in blood or serum was developed and marketed internationally, but especially in Germany and Switzerland. This test, named PrevoCheck, is based on the measurement of HPV16 major capsid protein L1‐specific monoclonal antibody clone DRH1. The authors suggest in their article that the assay can be used as a reliable screening tool for the early detection of HPV16‐induced cancers, including anogenital and oropharyngeal carcinomas [11]. In this study, we wanted to investigate the performance of PrevoCheck in a group of newly diagnosed HNSCC patients (HPV‐positive and HPV‐negative tumors) and compare the results of the test with the gold standard of p16 immunohistochemistry in combination with DNA PCR testing.

## 2 Materials and Methods

### 2.1 Patients and Study Design

This study was part of a larger cohort study involving a prospective collection of peripheral venous blood samples and was approved by the Medical Ethics Committee Kanton Zurich.

The study complies with the European Guidelines for Good Clinical Practice and written informed consent was obtained from each study participant. Eligible patients were patients with newly diagnosed histopathologically proven HNSCC with an age above 18 years. Exclusion criteria were patients with a HNSCC recurrence, patients with primary tumors of the salivary glands, thyroid glands, nasal cavity and paranasal sinuses, or patients without the cognitive ability to understand the information about the study. Between November 2021 and August 2022, 42 patients were included prior to treatment at the department of Otorhinolaryngology‐Head and Neck Surgery of the University Hospital Zurich, Zurich, Switzerland. We reviewed the medical records for detailed baseline demographic and clinical data on age, gender, smoking, and TNM stage (Table 1).

**TABLE 1:** Smoking was defined in pack years (1 pack year = 20 cigarettes a day for 1 year).

Characteristic	HPV‐positive *N* = 16 (38.1%)	HPV‐negative *N* = 26 (61.9%)	*p*
Gender			
Male	13 (81.3%)	19 (73.1%)	*p* = 0.72*
Female	3 (18.8%)	7 (26.9%)	
Mean age at diagnosis, years [range]	65.9 [55–85]	67.7 [40–84]	*p* = 0.60
Tumor subsite			
Oropharynx	16 (100%)	10 (38.5%)	*p* < 0.001
Other subsite	0	16 (61.5%)	
T‐stage			
cT1‐2	10 (62.5%)	15 (57.7%)	*p* = 0.76
cT3‐4	6 (37.5%)	11 (42.3%)	
N‐stage			
cN0	0 (0.0%)	17 (65.4%)	*p* < 0.001
cN1‐3	16 (100.0%)	9 (34.6%)	
Smoking			
Never (0–5 pack years)	8 (50.0%)	0 (0.0%)	*p* < 0.001
Yes (> 6 pack years)	8 (50%)	26 (100%)	

Abbreviations: HPV, human papilloma virus; no, number; PY, pack years.

* Fisher exact.

### 2.2 HPV Analysis

HPV testing was performed on all oropharyngeal tumors with the gold standard algorithm for HPV detection [7]. In short, immunohistochemical staining for p16INK4a was performed on 3 μm sections of formalin‐fixed paraffin‐embedded (FFPE) material according to the manufacturer's instructions. On the p16‐positive tumors (cut‐off point for p16‐positivity: 70% nuclear and cytoplasmic staining), high‐risk HPV‐DNA was detected with polymerase chain reaction (PCR). DNA was extracted from representative formalin‐fixed paraffin‐embedded (FFPE) tissue blocks, and standard primers PGMY09/11 and L1C1/2 were used. In case of low DNA quality, an alternative PCR protocol with GP5+/6+ primers was used. Specific HPV types were identified by sequencing analyses after purification from agarose gels. Internal (VCAN gene) and external (positive/negative) controls were run for each assay.

### 2.3 Performance of PrevoCheck

**FIGURE 1:**
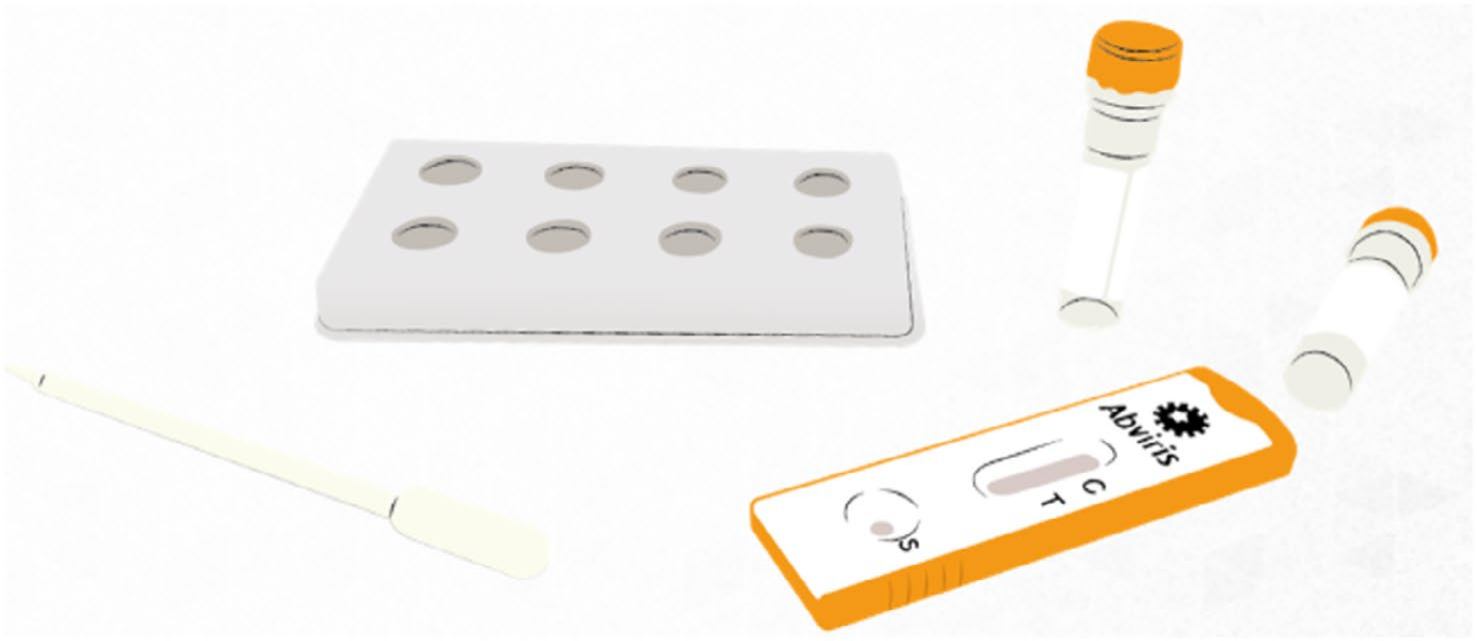
Materials of PrevoCheck.

**FIGURE 2:**
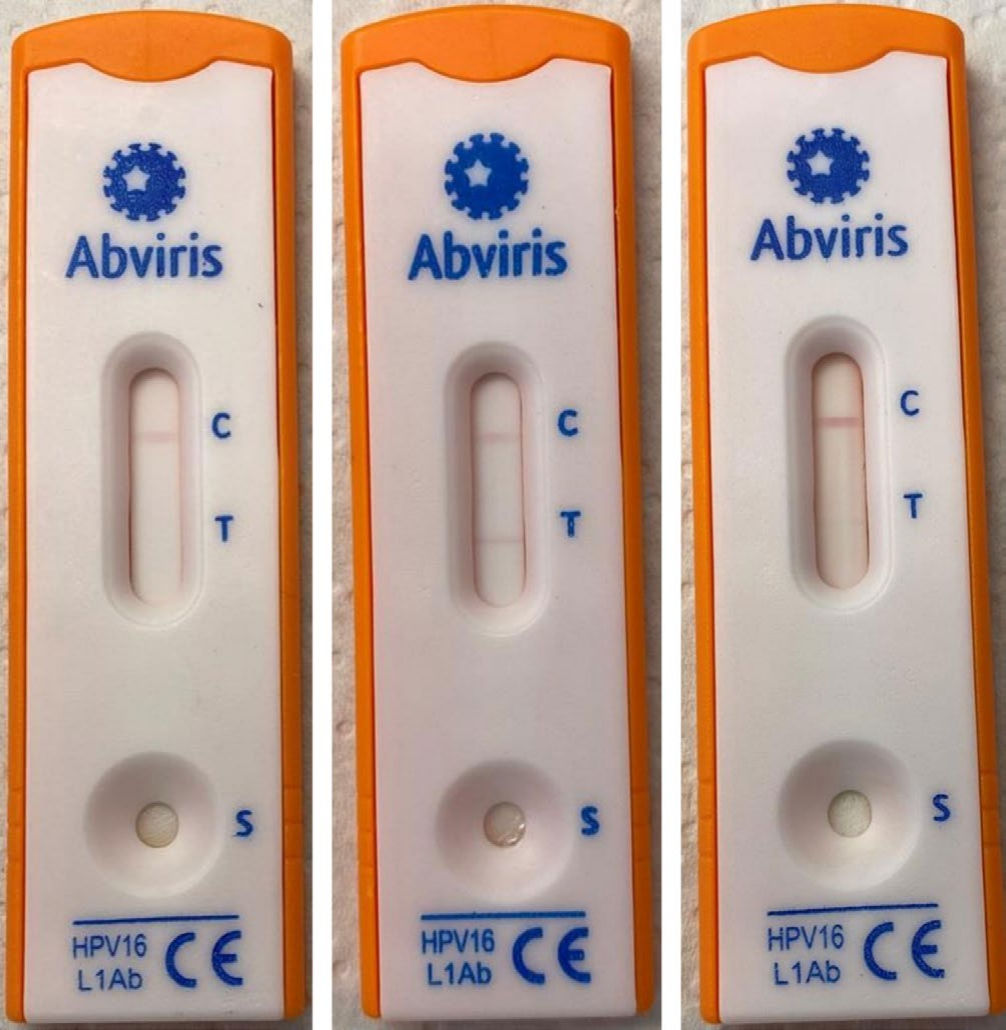
PrevoCheck result. (A) Negative result. (B) Positive result. (C) Equivocal result.

Venous blood samples were collected on the day of the first outpatient department visit in all patients using standardized collection devices and stored at 4°C until the samples were further analyzed. Serological detection of HPV16‐L1‐specific antibodies in whole blood or serum was performed on the same day the venous blood samples were taken, using the rapid PrevoCheck (Abviris, Germany), according to the manufacturer's instructions (Figure 1). After pre‐incubation of the blood sample with an HPV16‐L1‐specific reagent for 10 min, the mixture was transferred onto a lateral flow test cassette. Between 10 and 20 min later the test result was interpreted. The test is negative (i.e., normal result) when 2 lines appear (at the control (C)‐ and test (T)‐line) (Figure 2). The presence of a T‐line always indicates a negative result, even if the line is weak. The test is positive when only the C‐line appears (Figure 2). PrevoCheck is invalid when no lines appear or only the T‐line appears.

### 2.4 Data Analysis

The PrevoCheck result was interpreted by 2 reviewers with experience in head and neck oncology (C. Schouten [main reviewer] and T. Stadler). Reviewers were blinded to clinical information or HPV status at the time of interpreting the result. The observers interpreted the results individually and independently. All reviewers scored the results according to:

1. The manufacturer's instructions; a dichotomous system, that is, negative or positive.
2. A three point ordinal scale (negative, equivocal, and positive).

The three‐point scale was reduced to a binominal “sensitive interpretation” (equivocal and positive = a positive test result). The primary outcome was the accuracy (sensitivity, specificity, positive predictive value [PPV], and negative predictive value [NPV]) of PrevoCheck, in reference to the HPV status, defined as p16 positivity in immunohistochemical staining and detection of HPV DNA in tumor tissue. The secondary outcome was the interobserver agreement.

### 2.5 Statistical Analyses

Statistical analyses were performed using SPSS 28.0.0.0 software (IBM, Armonk, NY, USA). The level of significance was set at *p* < 0.05. Analysis for differences in patient characteristics between the HPV‐positive and HPV‐negative groups was performed with the Pearson Chi‐square test for categorical data and Fisher's exact test were appropriate. Exact 95% confidence intervals (CIs) were computed for the accuracy measures. Cohen's kappa and a 2 × 2 table were used to evaluate concordance between the observers [12]. The interpretations of kappa are: > 0: less than chance agreement, 0.01–0.19: slight, 0.20–0.39: fair, 0.40–0.59: moderate, 0.60–0.79: substantial, and 0.80–0.99: almost perfect agreement [13].

## 3 Results

### 3.1 Patient Characteristics

The total study group consisted of 32 men and 10 women, with a mean age at diagnosis of 66.4 years (range 40–85). Sixteen patients (38.1%) were diagnosed with an HPV‐positive HNSCC. In 13 of the 16 HPV‐positive tumors, HPV16‐DNA was detected. In the other 3 HPV‐positive cases, HPV35 or HPV69 was detected, and in one sample the DNA quality was too poor to perform DNA sequencing. Patient characteristics by HPV status are shown in Table 1. HPV‐negative patients were more likely to have a history of smoking. HPV‐positive patients were more likely to present oropharyngeal tumors and N‐positive disease.

### 3.2 Accuracy of PrevoCheck

Interpretation by the main reviewer according to the manufacturer's instructions showed 2 true positives, 26 true negatives, 0 false positives, and 14 false negatives (Table 2a). This resulted in a sensitivity of 12.5% (95% CI: 1.6%–38.4%) at a specificity of 100% (95% CI: 86.8%–100%) (Table 2b).

**TABLE 2a:** Results of PrevoCheck.

	PrevoCheck		
HPV		+	−
	+	2 (TP)	14 (FN)
	−	0 (FP)	26 (TN)

Abbreviations: FN, false negative; FP, false positive; HPV, human papillomavirus; TN, true negative; TP, true positive.

**TABLE 2b:** Diagnostic accuracy of PrevoCheck.

	PrevoCheck (*N* = 42)
Sensitivity % (95% CI)	12.5% (1.6–38.4)
Specificity % (95% CI)	100% (86.8–100)
PPV % (95% CI)	100% (15.8–100)
NPV % (95% CI)	65.1% (60.8–69.2)
Accuracy % (95% CI)	66.8% (50.5–80.5)

Abbreviations: NPV, negative predictive value; PPV, positive predictive value.

“Sensitive interpretation” of the PrevoCheck resulted in 5 true positives, 24 true negatives, 2 false positives, and 11 false negatives (Table 3a). This led to a sensitivity of 31.3% (95% CI: 11.0%–58.7%) at a specificity of 92.3% (95% CI: 74.9–99.1) (Table 3b).

**TABLE 3a:** Results of PrevoCheck “sensitive interpretation.”

	PrevoCheck (sensitive interpretation)		
HPV		+	−
	+	5 (TP)	11 (FN)
	−	2 (FP)	24 (TN)

Abbreviations: FN, false negative; FP, false positive; HPV, human papillomavirus; TN, true negative; TP, true positive.

**TABLE 3b:** Diagnostic accuracy of PrevoCheck “sensitive interpretation.”

	PrevoCheck (*N* = 42)
Sensitivity % (95% CI)	31.3% (11.0–58.7)
Specificity % (95% CI)	92.3% (74.9–99.1)
PPV % (95% CI)	71.4% (35.3–91.9)
NPV % (95% CI)	68.7% (60.7–75.3)
Accuracy % (95% CI)	69.1% (53.0–82.4)

Abbreviations: NPV, negative predictive value; PPV, positive predictive value.

Excluding the three HPV‐positive patients where HPV 16 was not detected resulted in 2 true positives, 26 true negatives, 0 false positives, and 11 false negatives. This led to a sensitivity of 15.4% (95% CI: 1.9%–45.5%) at a specificity of 100% (95% CI: 86.7–100).

Examples of negative, positive, and equivocal PrevoCheck results are shown in Figure 2. In all patients with a positive PrevoCheck (2 patients vs. 5 patients in “sensitive” reading), HPV type 16 was detected.

### 3.3 Interobserver Agreement

Analyses of the interobserver agreement on a dichotomous scale showed perfect agreement (kappa = 1.00) (Table 4a). With the “sensitive interpretation,” the interobserver agreement was almost perfect (0.81) (Table 4b).

**TABLE 4a:** Interobserver agreement for the dichotomous system.

		Obs 2		Kappa
		+	−	
Obs 1	+	2	0	1
	−	0	40	

*Note*: Values are presented as the number of patients and as kappa.

Abbreviation: obs, observer.

**TABLE 4b:** Interobserver agreement for the sensitive interpretation.

		Obs 2		Kappa
		+	−	
Obs 1	+	5	2	0.81 [0.46–1.0]
	−	0	35	

*Note*: Values are presented as the number of patients and as kappa with [95% confidence interval].

Abbreviation: obs, observer.

## 4 Discussion

Because many patients with HNSCC present with late‐stage disease, research is focused on the development of a reliable screening tool for the detection of HNSCC. Recent literature suggests that the level of serum antibodies to the HPV E6 and E7 oncoproteins is associated with HPV‐driven OPSCC [9]. The HPV genome consists of double‐stranded DNA and consists of seven early genes (E1–E7) and two late genes (L1 and L2). The oncoproteins E6 and E7 play a crucial role in oncogenesis, whereas L1 and L2 encode viral capsid proteins. Although PrevoCheck was developed to detect HPV16 L1 capsid antibodies, it was marketed that the test can be used to screen for HPV16‐induced cancers in the head and neck and anogenital area [11]. We studied the accuracy of the PrevoCheck in newly diagnosed HNSCC patients and found that the test resulted in a considerable amount of false negative results and had a poor sensitivity, varying from 12.5% to 31.3%. Obviously, a high sensitivity is the most important if the aim is to screen patients for HPV‐induced cancers, because a negative result in a test with a high sensitivity is useful for ruling out disease. According to our findings, this test does not seem accurate to rule out HPV16‐related head and neck cancers.

The usage of serum antibodies to HPV proteins as a potential screening tool for the detection of HPV‐related carcinomas has been investigated during recent years. HPV16 seropositivity occurs in response to an underlying HPV‐driven cancer and is expected to be low among healthy controls. In 2013, an association between HPV16 E6 antibodies (from blood samples collected at the time of cancer diagnosis) and HPV16‐related oropharyngeal cancer was reported [14]. In the years thereafter, it was shown that HPV seropositivity had a very high sensitivity and specificity for HPV‐related OPSCC [9, 15, 16].

Based on this high sensitivity and specificity, developing an early detection method using HPV 16 E6 antibodies could be useful. Kreimer et al. [17] reported that HPV16 E6 seropositivity was found in prediagnostic samples in 35% of patients with OPSCC. The increased risk of OPSCC in HPV16 seropositive patients was seen, ranging from less than 2 years until almost 14 years between serum collection and cancer diagnosis. In 2019, the HPV Cancer Cohort Consortium also demonstrated that HPV16‐E6 seropositive patients have a significantly increased risk of OPSCC. They showed that seroconversion can develop decades before OPSCC diagnosis but has a wide variation in the timing [18]. These findings implicate the usage of detecting HPV16‐E6 antibodies as a potential early screening method in clinical practice [19].

The association for other serum antibodies, interestingly HPV16 L1 seropositivity, with OPSCC was much weaker. Anantharaman et al. [14] found an odds ratio (OR) of developing oropharyngeal cancer when HPV16 L1 antibodies were detected of 8.60, whereas the OR of developing OPSCC when HPV16 E6 antibodies were detected was 132. This was also shown in the study by Kreimer et al. [17]. The OR for HPV16 L1 seropositivity was 3.1, in contrast to the OR for HPV16 E6 seropositivity, which was 274 for developing oropharyngeal cancer. Holzinger et al. [9] found a sensitivity of 53% and a specificity of 57% for HPV16 L1 antibodies, compared to a sensitivity of 96% and a specificity of 98% for HPV16 E6 antibodies.

The L1‐antibody response is considered unsuitable as a diagnostic screening tool since several studies have shown that the titers of HPV16 specific capsid antigens did not change after treatment [20, 21, 22]. It is thought that this antibody response represents cumulative past HPV infections from multiple anatomic sites. Seropositivity to the L1 capsid protein is an HPV lifetime exposure marker rather than a marker of acute HPV‐driven disease and can be long‐lasting. In addition, the L1‐antibody response reflects not only natural infections, as described in the previous paragraph, but it also reflects vaccine‐induced antibodies. A multicenter, double‐blind, randomized, placebo‐controlled trial showed a sustained immune response up to 4.5 years after vaccination [23]. This was shown as well in the study of Weiland et al. [11], where vaccinated people were included and these were all seropositive. The restriction that PrevoCheck should not be performed in vaccinated individuals is stated in the product manual, but we consider this an increasing disadvantage in using this test as a screening method. In conclusion, DRH1 does not seem to be suitable for detection of HPV‐driven OPSCC.

Conducting this study, we experienced that PrevoCheck is easy to perform and the test result is quickly acquired. However, we encountered two difficulties while carrying out this test. First, the intensity of the T‐line. The test should be interpreted as negative when both the C‐line and T‐line appear. The T‐line was often weak and weaker than the C‐line, and we were uncertain how to interpret the result of the test. Therefore, we included an equivocal test result in our analysis and analyzed the results sensitively. This “sensitive” reading did not improve the diagnostic accuracy of PrevoCheck. Second, the contrast between the test cassette and the T‐line. When we performed the test with whole blood, the membrane of the test cassette sometimes colored red, thereby reducing the contrast between the T‐line and the strip, making it more difficult to read the result. However, despite these difficulties, we did find a good interobserver agreement.

We acknowledge several limitations to this study. First, a limited number of patients were included, and the number of patients with HPV16‐driven oropharyngeal tumors was small. Nevertheless, we believe the study population is a reasonable reflection of the HNSCC patients who were newly diagnosed at the Zurich university hospital during the study time. Second, we did not perform HPV testing in non‐oropharyngeal tumors. Although the proportion of HPV‐positive oral cavity and laryngeal tumors increases, we feel that in this study the results would not have been different.

## 5 Conclusion

Although PrevoCheck is easy to perform and has a good interobserver agreement, the test resulted in a considerable amount of false‐negative results and the sensitivity for detecting HPV‐related OPSCC is poor. We do not recommend the usage of PrevoCheck as a screening tool for ruling out HPV‐induced OPSCC in the ENT or dental practice.

## Author Contributions

**Charlotte S. Schouten:** data curation, formal analysis, investigation, methodology, visualization, writing – original draft. **Vittoria Guarda:** data curation, investigation, methodology, writing – review and editing. **Thomas M. Stadler:** data curation, formal analysis, investigation, writing – review and editing. **J. Kristian Ikenberg:** data curation, writing – review and editing. **Martina A. Broglie Däppen:** conceptualization, methodology, project administration, supervision, writing – review and editing.

## Conflicts of Interest

Martina A. Broglie Däppen provides advisory services to MSD. The other authors declare no conflicts of interest.

## Data Availability

The datasets used and/or analyzed during the current study are available from the corresponding author on reasonable request.
